# An Atypical Presentation of Mycoplasma pneumoniae Infection Mimicking Acute Surgical Abdomen in an Adult

**DOI:** 10.7759/cureus.73665

**Published:** 2024-11-14

**Authors:** Jia Yi Lim, Timothy Wenham

**Affiliations:** 1 Internal Medicine, Barnsley Hospital NHS Foundation Trust, Barnsley, GBR; 2 Anaesthesia and Intensive Care Medicine, Barnsley Hospital NHS Foundation Trust, Barnsley, GBR

**Keywords:** acute abdomen, atypical presentation, extrapulmonary manifestation, mycoplasma pnemoniae, respiratory illnesses

## Abstract

*Mycoplasma pneumoniae* is a causative organism of atypical pneumonia and often presents with extrapulmonary symptoms and signs involving cardiovascular, dermatological, gastrointestinal and neurological systems. This is mostly seen in children but less commonly reported in adults. This case describes *M. pneumoniae* infection in an adult who presented with severe abdominal pain and was initially investigated for surgical causes and underwent diagnostic laparoscopy with unremarkable intraoperative findings. This case report highlights the importance of recognising atypical presentations of *M. pneumoniae* and consideration of non-gastrointestinal pathologies in patients presenting with acute abdominal pain.

## Introduction

*Mycoplasma pneumoniae-*associated pneumonia usually presents with vague constitutional symptoms rather than the classic upper and lower respiratory tract symptoms. This organism is also unique in which it lacks cell walls and hence is resistant to beta lactam antibiotics [1]. Even though traditionally *M. pneumoniae-associated* pneumonia is known to be mild and asymptomatic, it accounts for approximately 2-18% of community-acquired pneumonia [1]. Up to 25% of *M. pneumoniae* infections present with a wide array of extrapulmonary symptoms and they can present prior to, during, after or even without respiratory symptoms [2]. In the paediatric population, dermatological manifestations are the most common and can present as erythema nodosum, mucositis and Steven-Johnson syndrome [3]. There have been a few case reports detailing gastrointestinal manifestations including hepatitis and pancreatitis in the paediatric population [3-5]. Currently, there is scant evidence within the published literature detailing extrapulmonary manifestations of *M. pneumoniae-*associated infections in the adult population. This case report highlights an adult patient presenting with acute abdominal pain secondary to *M. pneumoniae* infection and aims to enrich this currently limited body of evidence.

This case report was presented as an e-poster at the 2024 World Congress of Internal Medicine (WCIM) from October 30 to November 2, 2024.

## Case presentation

A 22-year-old male patient presented acutely to the emergency department with right upper quadrant abdominal pain, vomiting, dysuria and fever. He had a background of well-controlled asthma, depression and a history of appendicectomy. Upon admission to the emergency department, he experienced severe pain requiring high degrees of analgesia including morphine. Aside from mild pyrexia, other initial observations were unremarkable including normal oxygen saturation. Observations on admission were as follows: respiratory rate of 22 breaths per minute, oxygen saturation of 99% under room air, blood pressure of 146/98 mmHg, heart rate of 100 beats per minute and temperature of 39 degree Celsius. The patient appeared to be slightly short of breath, but cardiovascular and respiratory examinations were within normal limits. However, the abdominal examination demonstrated right upper quadrant tenderness with localised guarding. His full blood count, urea and creatinine and liver function test were unremarkable although the C-reactive protein was raised at 60 mg/L and the lactate was 3 mmol/L (Table 1). The urine dipstick was negative for blood and leucocytes with 3+ ketones and 1+ protein. He was also initiated on intravenous co-amoxiclav as he was febrile and had a raised C-reactive protein pending further investigations. A referral was made to the surgical team to rule out conditions such as bowel perforation and adhesion colic.

**Table 1:** Laboratory investigations

Laboratory parameters	Results	Reference value
Haemoglobin	146 g/L	132-169 g/L
White blood cells	9.7 x 10^9^/L	3.7-10.0 x 10^9^/L
Neutrophils	7.4 x 10^9^/L	1.7-6.6 x 10^9^/L
Platelets	180 x 10^9^/L	150-450 x 10^9^/L
Amylase	32 U/L	30-118 U/L
Sodium	138 mmol/L	133-146 mmol/L
Potassium	3.5 mmol/L	3.5-5.3 mmol/L
Urea	3.3 mmol/L	2.5-7.8 mmol/L
Creatinine	81 µmol/L	53-97 µmol/L
Bilirubin	27 µmol/L	0-20 µmol/L
Alanine transferase (ALT)	16 U/L	10-49 U/L
Alkaline phosphatase (ALP)	60 U/L	30-130 U/L
Procalcitonin	0.19 ng/mL	<0.5 ng/mL
C-reactive protein	60 mg/L	<5 mg/L
Lactate	3 mmol/L	0.5-2.0 mmol/L

**Figure 1:**
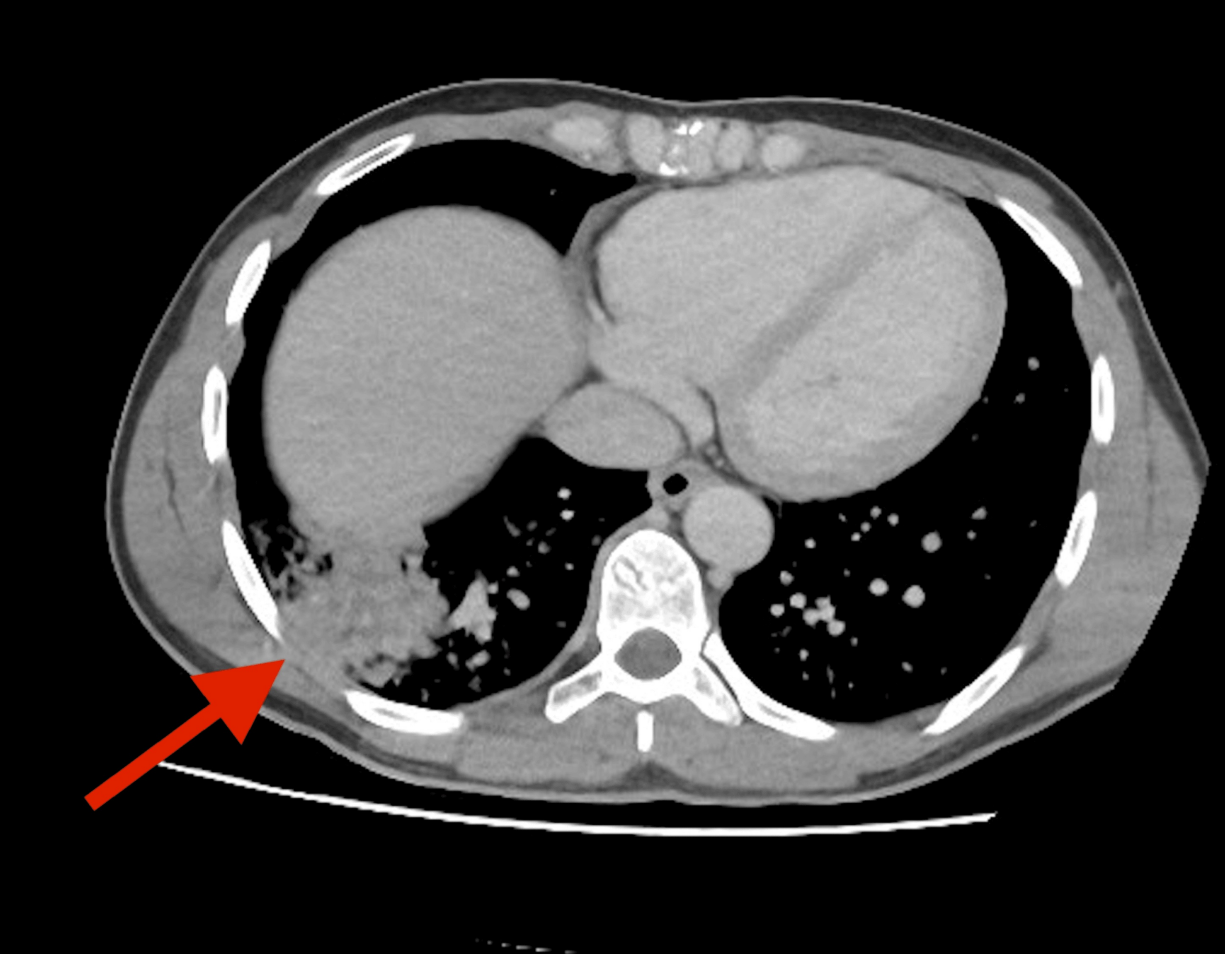
CT abdomen pelvis (axial plane) showing right lower lung consolidation

During his stay in the emergency department, the patient was found to have a syncopal episode with apnoea in which he recovered with supplementary oxygen. This was thought to be a vaso-vagal syncope secondary to severe abdominal pain. A CT of his abdomen and pelvis (Figure 1) was performed which demonstrated mild prominent small bowel loops suggestive of ileus and a right lower lung consolidation although there were no other pathologies that could account for his severe pain. Following administration of a total of 20mg of subcutaneous morphine to facilitate obtaining imaging, the patient suffered a respiratory arrest and was resuscitated after one cycle of CPR. The patient’s analgesic requirement continued to increase to the point of requiring intravenous ketamine and this worsening condition along with inconclusive CT findings prompted a decision by the surgical team to perform an exploratory laparoscopy for diagnostic purposes. The intraoperative findings were within normal limits and did not account for his abdominal pain.

Postoperatively, he was admitted to the intensive care unit for monitoring and pain optimisation. His abdominal pain remained difficult to control despite multiple different analgesia and he was initiated on patient-controlled analgesia intravenous oxycodone. The patient's condition worsened, with an increased oxygen requirement of five litres per minute via a nasal cannula. Further differentials were considered including sexually transmitted diseases and atypical infections. Screening tests for the above were carried out but returned negative. A respiratory PCR panel,* Legionella* and *Pneumococcal* urinary antigen tests were also performed in view of the lung consolidation and returned positive for *M. pneumoniae*. His antimicrobial therapy was then switched from co-amoxiclav to intravenous levofloxacin 500 mg twice a day after discussion with the microbiology team. However, he experienced vomiting from the administration of levofloxacin and his antimicrobial therapy was changed to oral clarithromycin 500 mg twice a day for seven days. His abdominal pain improved tremendously with the use of clarithromycin and was discharged two days later. The drastic improvement in the patient’s symptoms following a targeted switch in antibiotics suggests that the underlying aetiology for his abdominal pain was secondary to an *M. pneumoniae-*associated infection.

## Discussion

This case report highlights *M. pneumoniae* infection presenting initially with abdominal pain preceding other respiratory symptoms. This could be a manifestation of extrapulmonary symptoms of *M. pneumoniae* infection as it is known to be associated with non-specific gastrointestinal symptoms such as nausea and vomiting. There are also case reports on *M. pneumoniae-*associated pancreatitis and hepatitis although these are not evident in this case [3-5]. Although the pathophysiology of extrapulmonary manifestations has not yet been fully elucidated, three possible mechanisms have been proposed, namely, the direct bacterial inflammatory response, an indirect autoimmune response and vascular occlusion [6,7]. 

The other plausible explanation for the abdominal pain is a result of referred pain from the right lower lobe of the lungs. In the present case, it is postulated that the pleural inflammation of the right lower lobe and diaphragm irritation resulted in the stimulation of the intercostal nerves which subsequently manifested as referred right upper quadrant abdominal pain. This mechanism has also been described by Lei et al. in the case of a paediatric patient with *M. pneumoniae* infection presenting with acute abdominal pain and scrotal swelling [8]. Abdominal pain is a common presentation of pneumonia in the paediatric population but much less commonly seen in adults. The rare presentation of extreme abdominal pain as the sole symptom of pneumonia in adults is described by Naccour et al. in which a patient with community-acquired pneumonia was initially diagnosed as surgical abdomen and underwent diagnostic laparoscopy [9].

The diagnostic methods for *M. pneumoniae* can be broadly divided into serology, culture and polymerase chain reaction (PCR) [2,10]. Traditionally, *M. pneumoniae* can be detected by cold agglutinin titres [10]. However, this test has low sensitivity and can be positive in other infections such as cytomegalovirus and Epstein-Barr virus [10]. Other more advanced serological tests include complement fixation, microparticle agglutination assay (MAG) and ELISA [10]. Culture is not routinely used in clinical practice as isolation of *M. pneumoniae* is expensive, slow and not widely available [2,10]. Standard PCR is the preferred diagnostic method due to its rapid results, high sensitivity and specificity as well as being useful in the detection of pathogens in extrapulmonary sites [2,10].

**Figure 2:**
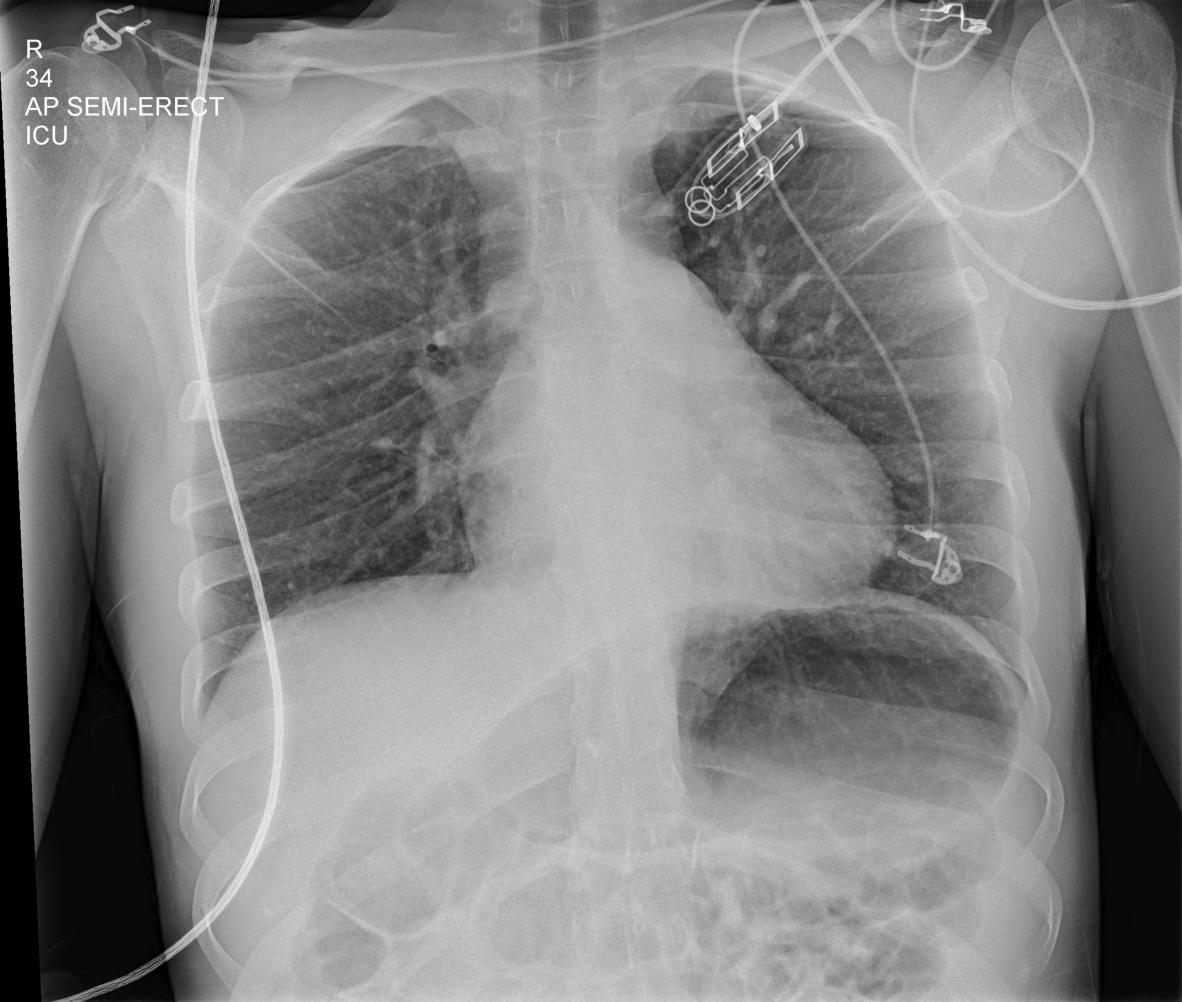
Chest X-ray showing clear lung fields

Aside from the variability in clinical presentation, there is also a lack of uniformity in radiological features associated with *M. pneumoniae* infection. Amantea et al. described five cases of *M. pneumoniae* infections all with different radiological features and none of them were considered to have *M. pneumoniae* infection initially [11]. In our case, a chest X-ray (Figure 2) was performed after the abdominal CT failed to pick up on the consolidation that was described in the CT scan. This showed that plain chest radiographs are not the best modality to assess atypical pneumonia. The variable presentation and lack of identifiable features on plain chest radiographs also add to the challenge of diagnosing *M. pneumoniae* infection, further causing a delay in diagnosis.

Treatment for extrapulmonary manifestations of *M. pneumoniae* infection is still lacking in evidence. Macrolides are the mainstay as tetracyclines and fluoroquinolones can cause significant dental and skeletal side effects in children [3]. The role of steroids in severe *M. pneumoniae* pneumonia is evident in a case series of 15 children who were non-responsive to macrolides but responded well to a course of prednisolone treatment [12]. There is also evidence detailing the use of steroids, plasmapheresis and intravenous immunoglobulins in *M. pneumoniae *infections with severe neurological manifestations such as transverse myelitis and encephalomyelitis [2,13]. However, with the lack of concrete understanding of pathology underlying *M. pneumoniae* infection, treatment modalities could yet to be improved in the future.

## Conclusions

This case demonstrates that *M. pneumoniae *infection can present in various forms and the primary symptoms might not be confined to the respiratory system. Although more commonly seen in the paediatric populations, extrapulmonary symptoms can present in the adult population as well. Clinicians should always keep a broad differential diagnosis while managing acute abdominal pain as it could be a manifestation of other non-gastrointestinal diseases. A good understanding of pain innervation and transmission pathway, alongside careful physical examination and detailed investigations, could lead to prompt and accurate diagnosis. Early recognition of the infection and switching treatment to the right antimicrobials could avoid unnecessary investigations and improve patient care.
